# The surprising influence of family history to type 2 diabetes on anaerobic performance of young male élite athletes

**DOI:** 10.1186/2193-1801-3-224

**Published:** 2014-05-03

**Authors:** Antonino Bianco, Francesco Pomara, Antonino Patti, Ewan Thomas, Marco Petrucci, Marianna Bellafiore, Giuseppe Battaglia, Antonio Paoli, Antonio Palma

**Affiliations:** Sport and Exercise Sciences Research Unit, University of Palermo, Via Eleonora Duse 2, 90146 Palermo, Italy; MEDEOR Research Institute, Palermo, Italy; Department of Biomedical Science, University of Padua, Padova, Italy

**Keywords:** Type 2 diabetes, Anaerobic performance, Élite athletes, Family history, Wingate test, Track and field, Performance

## Abstract

**Aims/Hypothesis:**

It is known that family history to type 2 diabetes induces anthropometric changes in various populations. Regular physical activity can induce adaptations in these subjects regularizing body composition and anthropometric parameters. The aim of this study is therefore to understand if family history to type 2 diabetes affects anaerobic performance in young male élite athletes.

**Methods:**

Forty six young male élite athletes were tested. Thirty three without family history to type 2 diabetes (FH-) and thirteen with family history to type 2 diabetes (FH+). Anthropometric parameters, body composition, physiological parameters and athletic performance were assessed.

**Results:**

Weight (*p* 0,0050), BMI (*p* 0,0019), waist circumference (p 0,0090), hips circumference (p 0,0490) and WHR (*p* 0,0339) were different between the two groups, showing greater values for the FH + subjects. Body composition showed lower FM and higher FFM percentages for the FH + group compared to the FH-. Anaerobic performance tests showed differences between the groups highlighting that the FH + group had higher anaerobic performance values (Wingate test for FH + 512,77 ± 107,93 W *vs* Wingate test for FH- 447,94 ± 56,95 W).

**Conclusions/Interpretation:**

The élite athletes with FH + showed better anaerobic performances and a higher body mass. At this stage we cannot generalise, but in a sample of athletes we tested, all who were with FH + showed both interesting and unexpected results; if confirmed, this evidence may represent a remarkable knowledge for fitness coaches and professionals who are daily dealing with track and field athletes and their performances.

## Introduction

In occidental industrialized countries, type 2 diabetes (T2D) is a widespread disease, showing a constant increase of its incidence also in a young population (Bianco et al. 2013). Moreover even genetic and environmental risk factors seem to influence T2D development: family history, age, obesity and physical inactivity are the major (Mahajan et al. 2013). The hereditary mechanisms of T2D have been also demonstrated in women with positive family history (FH) to the illness confirming the inter-generative transmission of this disease (Erasmus et al. 2001;Crispim et al. 2006;Rodekamp et al. 2005;Plagemann et al. 2002;[Bibr CR4][Bibr CR5];Grill et al. 1999;Rosenbaum et al. 2013). Several Authors have also showed that FH to diabetes has an influence on metabolic and anthropometric characteristics ([Bibr CR1][Bibr CR3]). BMI, weight and hip-waist ratio, have been showed to be greater in subjects with FH of T2D compared to those who were not (Pomara et al. 2006). Although, physical activity has been shown to be preventive on the onset of T2D and can be a useful strategy in reducing firstly abdominal obesity and secondly BMI (Kim and Lee 2009;Golubic et al. 2014). There are also strong evidence shown in scientific literature underlining that physical activity has a role on normalizing glucose metabolism and body composition. Has shown by Bianco et al. regular physical activity in subjects with family history to this illness can normalize the above mentioned parameters and make them comparable to those of subjects with no FH (Bianco et al. 2011). Moreover increases in aerobic activities improve cardiorespiratory fitness and cardiovascular risk factors and are useful in stabilizing plasma glucose, body composition and insulin resistance (Geidl and Pfeifer 2011;O’Hagan et al. 2013). Other important considerations must be made for anaerobic activities that are also known to be able in improving insulin sensitivity and glucose tolerance (Kourtoglou 2011;Mann et al. 2013) and as a consequence of the practiced activity, increase muscle mass, strength and reduce total fat mass (Kwon et al. 2010). Though, little is still known about the relationship between family history to T2D and physical performance, therefore the objective of this study is to understand how anaerobic performance is affected by family history to T2D in young male élite athletes.

## Materials and methods

### Subjects

Forty six young male élite athletes practicing track and field were tested. Thirty three (age 23,15 ± 5,14 years, height 171,30 ± 7,54 cm, weight 64,71 ± 9,01 kg, 7 endurance athletes and 26 power athletes) did not present FH to T2D while thirteen (age 25,31 ± 4,21 years, height 174,65 ± 8,94 cm, weight 76,42 ± 17,92 kg, 3 endurance athletes and 10 power athletes) did. The subjects were recruited according to our inclusion criteria. They had to be 1) involved in track and field activities from at least 5 years, 2) be involved in competitions and 3) were not using any medication or not suffering from any apparent illness. Anthropometric parameters, body composition, physiological parameters and athletic performance were assessed. Each subject completed a questionnaire in order to determine physical, medical and nutritional history. A standard questionnaire was subsequently administered to assess FH to T2D: subjects with at least a sibling or parent with the illness were coded as “high-degree” (FH+) while subjects without family members with the illness were coded as “with no-familiarity” (FH-); The athletes were enrolled in the study according to the inclusion criteria approved by the ethics committee of the University of Palermo. The principles of the Italian data protection act (196/2003) were observed. All participants provided informed consent. The study was performed in compliance with the Helsinki Declaration.

### Anthropometry and body composition

Weight was measured to the nearest 0.1 kg with a calibrated physician’s scale (Seca 709, Hamburg, Germany), and height to nearest 1 mm with a wall-mounted stadiometer (Seca 220, Hamburg, Germany). Waist circumference and hip circumference were measured to the nearest 1 mm, and subsequently the waist-hip ratio (WHR) was calculated. All measurements were performed by the same investigator. Body mass index (BMI) was also calculated. Body composition as free-fat mass (FFM) and fat mass (FM) were assessed by whole body impedance analysis at 50 kHz using a bio-electrical analyser (BIA, model 109, RJL Systems, Detroit - MI) following manufacturer’s recommended protocol. Patients were previously instructed to avoid alcohol intake and exhausting physical activity on the day before the exam and to limit food or fluid intake to 4 h before the test.

### Physiological parameters

Basal metabolic rate (BMR), VO_2_ Max, Heart rate (HR) and blood lactate (BL) were assessed. BMR was measured inside a quiet and comfortable darkened room, with a temperature between 22 and 24°C, in the morning between 8:30 and 10:30, following an overnight fast and a standardised meal consumed before 9:00 pm of the preceding evening. Subjects were euhydrated and had refrained from exercise for at least 36 h. After a habituation period of 10–15 min, BMR was recorded continuously for at least 30 min by an open-circuit indirect calorimetry in dilution testing mode (Vmax 29 N Sensormedics Italia Srl, Milano, Italy), using a perspex canopy. Oxygen was measured using a gas analyser, on the basis of the high paramagnetic susceptibility of oxygen; carbon dioxide levels were determined by an analyzer using the rapid-Response Non-Dispersive Infrared (NDIR) measurement technique. Values were averaged over 1-min intervals and recorded in a raw data file. BMR was expressed in Kcal per 24 hr (Kcal/day), VO_2_ Max was measured telemetrically using an oxygen analyser (K4B2, Cosmed, Italy). The laboratory experiments were conducted under standard conditions of temperature and humidity. HR was monitored using a Polar^®^ Heart rate monitor (wireless double electrode). VO2 Max and HR were measured during an incremental treadmill test until muscle exhaustion. VO2 Max was expressed in absolute values (L/min) and HR in beats per minute (bpm). BL was analyzed using an Accutrend Lactate photometric test in vitro, Roche Diagnostic GmbH, Germany. For each subject BL was recorded at regular intervals: basal values before the tests and 2, 4, 6 and 8 minutes after anaerobic testing, during recovery.

### Athletic performance

Each subject had to perform a squat jump and a Wingate test. The squat test was performed squatting down until the knees were bent at 90 degrees, while swinging the arms back behind the body. Without pausing, the arms were swung forwards and the athletes had to jump as high as possible, landing back on the mat on both feet at the same time. The best of three attempts was took in consideration. Height and flying time were recorded trough a BOSCO platform (Byomedic, S.C.P., Barcelona, Spain).

Subsequently, after a 5 minute rest, each subject had to perform a cycle Wingate test trough a cycle sonar Ergomedic 894E Peak Bike (Germany). After a low intensity warm up, athletes started to cycle at maximal speed without any resistance. After three seconds a researcher inserted a standard resistance (75 g/kg of weight); Athletes had to cycle for 30 seconds at maximal velocity; a software recorded and stored resistance and cycle velocity variations during the test. Peek power output was then used for statistical analysis.

### Statistical analysis

All data were recorded on an Excel file for Windows. Differences between the two groups (FH + and FH-) were assessed using an unpaired two tailed *t* test. Data processing was performed using Statistica 8.0 for Windows (Statsoft Inc., Tulsa, OK, USA). Significance level was set at *p* < 0.05 for all analyses.

## Results

Significant differences were shown for anthropometric parameters between groups. Weight (*p* 0,0050), BMI (*p* 0,0019), waist circumference (p 0,0090), hip circumference (p 0,049), WHR (*p* 0,0339), showing greater values for the FH + subjects (Table 1).

**Table 1:** **Participants’ characteristics**

Characteristic	FH- (***n*** = 33)	FH + (***n*** = 13)	***p*** value
Age (years)	23,15 ± 5,14	25,31 ± 4,21	ns
Height (cm)	171,30 ± 7,54	174,65 ± 8,94	ns
Weight (kg)	64,71 ± 9,01	76,42 ± 17,92	0.0050
BMI (kg/m^2^)	21,96 ± 1,88	24,76 ± 3,90	0,0019
WHR	0,94 ± 0,05	0,97 ± 0,03	0,0262

No differences were found regarding any of the physiological parameters, although the FH + group showed higher BMR and lower percentage values of FM (18,74 ± 5,69%) compared to the FH- (19,90 ± 5,77%) even if not statistically different (*p* 0,189) and higher FFM (81,26 ± 5,69) compared to the FH- (80,10 ± 5,77) (p 0,0040) (Table 2).

**Table 2:** **Physiological parameters**

	FH- (***n*** = 33)	FH + (***n*** = 13)	***p*** value
BMR (calories/day)	1450 ± 23,71	1619 ± 73,06	0,0501
VO_2max_ (ml/kg/min^-1^)	4112 ± 175,1	4413 ± 215,4	0,3259
MHR (beats/min)	188,5 ± 1,634	189,0 ± 3,017	0,8838
BL (mmol/l) 2 min rest	10,12 ± 0,8934	11,24 ± 1,789	0,5377
BL (mmol/l) 4 min rest	9,838 ± 0,6484	10,04 ± 0,124	0,9644
BL (mmol/l) 6 min rest	8,746 ± 0,5906	9,903 ± 0,8621	0,4168
BL (mmol/l) 8 min rest	7,371 ± 0,6143	8,249 ± 0,8072	0,4378

**Figure 1:**
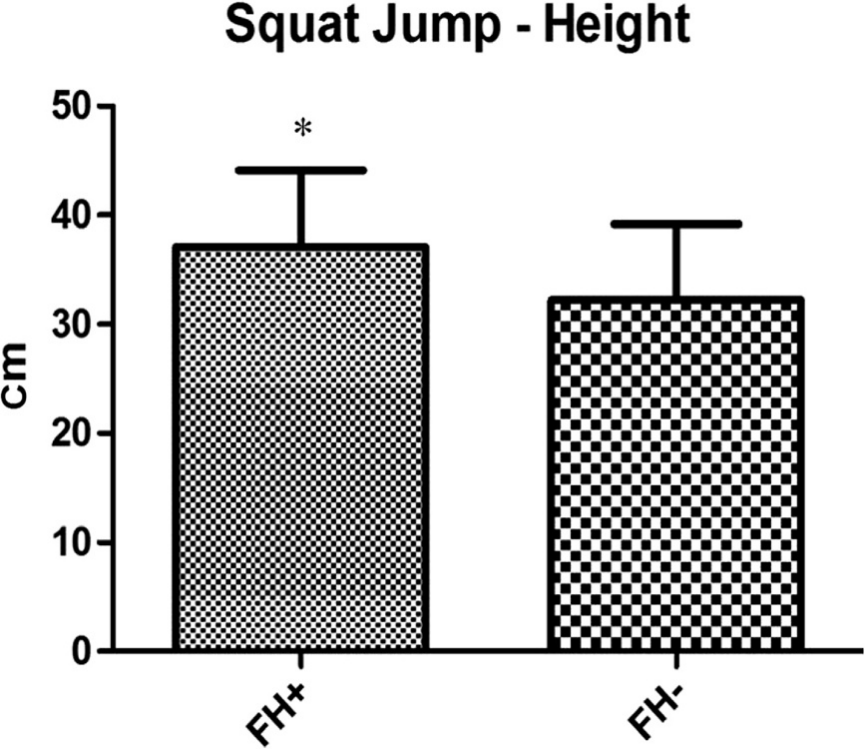
**The figure is showing the squat jump performances of both groups.**

**Figure 2:**
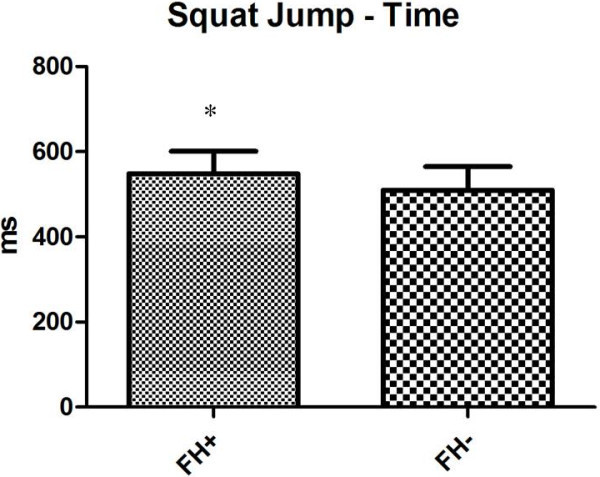
**The figure represents the squat jump time of flight of both groups.**

**Figure 3:**
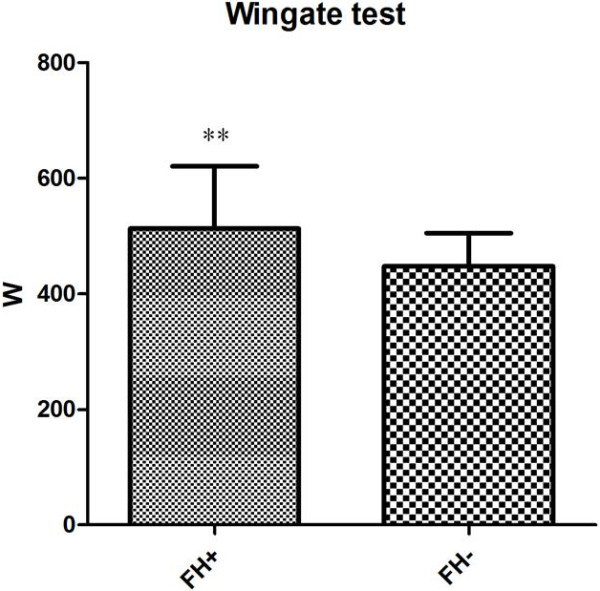
**In this figure, the Wingate tests of both groups are present.**

Regarding anaerobic performance both tests showed significant differences. The squat jump of the FH + group was greater than the FH- both in height (37,12 ± 7 cm vs. 33,23 ± 6,95 cm, respectively) (*p* 0,0379) and flying time (548,15 ± 52,89 ms vs. 509,82 ± 55,01 ms, respectively) (*p* 0,0370) as shown in Figures 1, 2 as also the Wingate test (512,77 ± 107,93 W vs. 447,94 ± 56,95 W, for FH + and FH-, respectively) (*p* 0,0116) as shown in Figure 3. The results highlight that the FH + group show to some extent higher anaerobic performance values (Table 3).

**Table 3:** **Athletic performance with significant difference (** ***p*** **< 0,05)**

Athletic performance	FH- (***n*** = 33)	FH + (***n*** = 13)	***p*** value
Squat jump-height (cm)	33,23 ± 6,95	37,12 ± 7	0,0379
Squat jump-flying time (ms)	509,82 ± 55,01	548,15 ± 52,89	0,0370
Wingate test (W)	447,94 ± 56,95	512,77 ± 107,93	0,0116

## Discussion

The study results are in line with scientific literature, showing higher values in subjects with FH to T2D (Bianco et al. 2011,2014). In our previous study we analyzed whether physical activity could influence basal metabolic rate in sedentary and active women with FH to T2D and the results show that active women, had similar basal metabolic rates compared to those with no FH to T2D, despite this familiarity to the illness; on that case physical activity played a key role on a FFM and BMR maintenance (Bianco et al. 2011). In this study élite athletes were compared; this particular sample (élite athletes) allows us to state that the differences found between the groups could not be determined by differences in their physical level. The only independent variable of our sample is in fact FH to T2D. Although, it has been noticed that the majorities of studies regarding FH to T2D mainly investigate on the effects of aerobic exercise on health (Ekelund et al. 2007; Nyholm et al. 2004), regarding subjects with FH to T2D little was known about the relationship of this hereditary profile with physical performance. Moreover, the choice of the Wingate test for the assessment of peak power output was not accidental; though Hawley and Gibala don’t recommend the use of “all out” tests in a clinical population (Hawley and Gibala 2012), the strong physical conditioning to which these athletes undergo has allowed us to administer this test. The results show higher anaerobic peak power outputs in the FH + group compared to the FH-.

Scientific literature has recently started studying anaerobic exercise interventions on subjects with T2D and the main outcomes show that these type of activities help reducing fat mass, elicit strength and prevent lean mass loss (Kwon et al. 2010). Anaerobic activities also help enhancing the uptake of glucose in the muscle (Christ-Roberts et al. 2003; Mann et al. 2013; Gordon et al. 2013) and have a role in treating bone dysfunctions in this particular clinical population (Wood and O’Neill 2012). Recently, Nitert et al. published an interesting study on impact of an exercise intervention on DNA methylation in skeletal muscle from first-degree relatives of patients with type 2 diabetes. The Authors identified epigenetic differences in muscle of FH + compared with FH- individuals. In more details, the differential DNA methylation of genes in biological pathways with key functions in muscle such as MAPK, insulin, and calcium signaling and of individual genes including PRKAB1 and MAPK1 (Nitert et al. 2012). It is demonstrated that MAPK1 have an important physiological and metabolic roles in skeletal muscle (Wojtaszewski et al. 1999), moreover the protein encoded by PRKAB1 is a regulatory subunit of AMP-activated protein kinase, which is an enzyme that monitors cellular energy status and regulates metabolism in muscle (Hardie 2008). Nitert et al. demonstrate that exercise for 6 months is associated with decreased DNA methylation of MEF2A (Nitert et al. 2012). The MEF2A (Myocyte-specific Enhancer Factor 2A) is a transcription factor involved in the exercise-induced regulation of GLUT4 expression, and hence it may influence glucose uptake in muscle (McGee et al. 2006).

Moreover still in Nitert study a number of genes changed their expression after training period (all related to the muscle metabolism regulation) (Nitert et al. 2012). Those epigenetic differences seem to be related to a combination of FH to T2D with a long term practice of sport activities. This should be one possible explanation to a better anaerobic performance and higher FFM of our FH + participants.

## Conclusion

The evidences that are coming out from this study are both interesting and unexpected. The élite athletes with FH toT2D showed better anaerobic performances and a higher body mass. This evidence seems to be supported by few observational studies carried out by our research group and few clinical studies who deeper investigated the biological mechanisms responsible of phenotype mutation and epigenetic differentiation in people with FH toT2D. More studies with a larger number of participants are needed in order to consolidate this first study. At this stage we cannot generalise, but in a sample of athletes we tested, all who were with FH showed a significant better anaerobic performance; if confirmed, this evidence may represent an interesting knowledge for fitness coaches and professionals who are daily dealing with track and field athletes and their performances.

## Abbreviations

BL: Blood lactate
BMR: Basal metabolic rate
FFM: Free-fat mass
FH: Family history
FH-: Subjects without family history to type 2 diabetes
FH+: Subjects with family history to type 2 diabetes
FM: Fat mass
HR: Heart rate
RNDI: Response Non-Dispersive Infrared
T2D: Type 2 diabetes
VO2Max: Maximal oxygen uptake.
